# Relations between trajectories of weight loss and changes in psychological health over a period of 2 years following bariatric metabolic surgery

**DOI:** 10.1007/s11136-025-03906-1

**Published:** 2025-01-29

**Authors:** Johanna Eveliina Pyykkö, Nienke van Olst, Victor E. A. Gerdes, Josué Almansa, Yaïr I. Z. Acherman, Maurits De Brauw, Albert K. Groen, Max Nieuwdorp, Robbert Sanderman, Mariët Hagedoorn

**Affiliations:** 1 Department of Health Psychology, Faculty of Medical Sciences, University of Groningen, University Medical Center Groningen, Antonius Deusinglaan 1, 9713 AV Groningen, The Netherlands; 2 Department of Metabolic and Bariatric Surgery, Spaarne Gasthuis, Hoofddorp, The Netherlands; 3 Department of Internal Medicine, Spaarne Gasthuis, Hoofddorp, The Netherlands; 4 Department of Vascular Medicine, Amsterdam UMC, Amsterdam, The Netherlands; 5 Division of Community and Occupational Medicine, Department of Health Sciences, University Medical Center Groningen, University of Groningen, Groningen, The Netherlands; 6 Department of Experimental Vascular Medicine, Amsterdam UMC, Amsterdam, The Netherlands

**Keywords:** Bariatric metabolic surgery, Obesity, Health-related quality of life, Depression, Weight, Trajectories

## Abstract

**Purpose:**

This study aimed to identify trajectories of BMI, obesity-specific health-related quality of life (HR-QoL), and depression trajectories from pre-surgery to 24 months post-bariatric metabolic surgery (BMS), and explore their associations, addressing subgroup differences often hidden in group-level analyses.

**Method:**

Patients with severe obesity (*n* = 529) reported their HR-QoL and depression before undergoing BMS, and at 12 and 24 months post-operation. Latent Class Growth Analysis was used to identify trajectories of BMI, HR-QoL and depression.

**Results:**

BMI and HR-QoL improved significantly for all patients from pre-surgery to 24 months post-operation, though some patients deteriorated in their outcomes after 12 months. Three distinct trajectories of BMI were identified: Low (35.4%), Medium (45.5%), and High (19.2%), and of HR-QoL: High (38.4%), Medium (43.4%), and Poor (18.1%). Three trajectories of depression were extracted: Low/none (32.4%), Medium–low (45.3%), and Worsening (22.3%). The association between the trajectories of BMI and depression was significant, but not between the BMI and HR-QoL trajectories. Specifically, the Low BMI trajectory patients were more likely to follow the Worsening depression trajectory and reported poorer preoperative psychological health than the other two BMI trajectories.

**Conclusion:**

Patients following the most favourable weight loss trajectory may not manifest psychologically favourable outcomes (i.e., Worsening depression), and preoperative characteristics do not consistently describe post-surgical BMI trajectories. Clinicians should tend to patients’ mental wellbeing besides weight loss post-BMS. The study findings emphasize the significance of incorporating psychological health as an essential component of surgical outcomes.

## Introduction

Bariatric metabolic surgery (BMS) is an effective treatment method for severe obesity, leading to substantial weight loss and amelioration or even complete resolution of obesity-related medical conditions such as cardiovascular disease and diabetes [1–3]. Mean weight loss is approximately 30% 1 year after the surgery and 25% 15 years after the surgery [2–4]. The individual outcomes vary greatly, as a minority of patients (approximately 5%) start to regain weight as early as 6 months after surgery, whereas others start to regain body mass after reaching their lowest weight (‘nadir’), which usually occurs within the first 2 years after the surgery [3–6]. Diverse definitions of weight regain are currently found in the literature, such as the reacquisition of 10 kg or more, exceeding a Body Mass Index (BMI) of 35 kg/m^2^, or surpassing specific thresholds relative to presurgery, nadir or maximum weight loss, for example an increase of bodyweight exceeding 10, 20, or 25%, all indicative of clinically significant weight regain [7–9]. Most patients experience some weight regain after undergoing BMS, and anatomical, behavioural and psychological factors are thought to be the reason for weight gain exceeding initial expectations [10–12].

Recent evidence suggests that response to surgical treatment does not only vary between patients, but that distinct weight loss trajectories can be observed among patients after BMS [5, 6, 13–17]. Researchers have identified up to six different weight loss trajectories during the first seven post-operative years, differing in their speed and magnitude of weight loss and regain [5, 6, 13–17]. Further, different weight loss trajectories have been associated with demographic, personality and psychopathological characteristics [5, 13, 14], and even predicted by postoperative problematic eating behaviours [18].

Furthermore, patients’ health-related quality of life (HR-QoL) and depressive symptoms have been shown to improve after BMS [19–22]. Improvement of psychological health is an important surgical outcome but also an important motivator for surgery [23]. Recent studies have identified various trajectories in mental and physical HR-QoL among patients, with most presenting sustained improvement up to 3 years post-surgery [17, 24]. The changes in HR-QoL coincide with changes in postsurgical weight [25, 26]. Moreover, the changes in HR-QoL trajectories were associated with small variations in psychological wellbeing. For example, higher BMI, anxiety, binge eating and depression were associated with a decline in mental and physical HR-QoL in some of the HR-QoL trajectories [24]. Much of the research up to now has been limited to using generic HR-QoL measures that are less sensitive to the clinical aspects of HR-QoL experienced by individuals with obesity, and to focusing on the net change in HR-QoL after BMS instead of investigating patterns and stability of change [22, 27, 28].

A well-established, complex relationship exists between obesity and psychological wellbeing. Namely, obesity can increase the vulnerability for mental health issues, and conversely, mental health issues can predispose to obesity [29, 30]. Individuals with overweight had 55% higher risk to develop depression, and depression increased the risk of developing excess weight by 58% [30]. Increasing body weight has been linked to diminished body image and self-esteem, which are crucial components of life satisfaction and HR-QoL [31–34]. Self-efficacy, which refers to the belief in one’s capability to execute a behaviour to attain a specific goal or outcome, is a significant predictor of actual behaviour, for example exercise and nutritional intake [35–37]. Recently, research focus has expanded to examine how psychological traits, such as attachment style, personality, social support, influence individual’s health behaviours and outcomes [38–40]. Identifying potential preoperative characteristics linked to different outcome trajectories could facilitate our understanding of patient needs and guide the development and offering of treatment plans.

Studies investigating associations between psycho-behavioural characteristics and weight trajectories following BMS have been limited to smaller sample sizes (i.e., *n* < 100), retrospective data, and comparing trajectories between different patient groups (e.g. surgical vs non-surgical, primary vs re-operative patients) [14, 16, 18, 26, 41]. Thus, knowledge of psycho-behavioural factors associated with different trajectories of weight loss and psychological outcomes is scarce. Furthermore, the group characteristics and psychological factors linked to the different post-surgical HR-QoL and weight loss trajectories are poorly understood. Analysing the patterns of weight loss and psychological health following BMS allows for an evaluation of the relationship between changes in physical and psychological health. This analysis enables the detection of key points in time requiring additional support, the identification of potential factors linked to unfavourable surgical outcomes, and the early recognition of suboptimal weight loss trajectories. Moreover, this comprehensive understanding can contribute to optimizing post-surgical interventions by refining their content and timing, ultimately enhancing treatment outcomes.

The purpose of this investigation was to identify and describe clinically distinct trajectories of three important outcomes for bariatric metabolic surgery; namely, weight (measured as BMI), obesity-specific HR-QoL, and depression from pre-surgery to 24 months after surgery. We expected to find distinguishable outcome trajectories in our sample with either constant improvement, first improvement and then stabilization, or first improving then worsening trajectories. Secondly, we aimed to examine the associations between the different trajectories, as we expected the changes in psychological health to match the changes in BMI. Third, we sought to provide data on the association between the trajectory class membership and preoperative psychological (attachment style, body image, food cravings, and self-efficacy for controlling eating behaviour) and demographic factors (i.e., age, sex).

## Method

### Design, participants and procedure

Available data from two larger ongoing studies, namely the BARIA- [42] and DIABAR-studies [43], were used in the current analyses. Participants were recruited from the Medical Center Slotervaart in Amsterdam, and Spaarne Gasthuis Hoofddorp, the Netherlands. Eligibility criteria include a BMI above 40 kg/m^2^ or a BMI over 35 kg/m^2^ with obesity-related comorbidity, age between 18 and 65 years, no previous BMS, and a previous attempt to lose weight under supervision. Patients were assessed at the hospital approximately 30 days prior to the surgical operation, and 12 and 24 months after the surgery. Most patients underwent a laparoscopic Roux-en-Y gastric bypass. However, if a patient had a strong preference or the surgeon recommended another method based on patient’s clinical characteristics, a laparoscopic omega loop gastric bypass or sleeve gastrectomy was performed. These surgical methods produce comparable outcomes in terms of weight loss, improvement in comorbidities, and psychological health [44, 45]. For the current analyses, data collected from 1st September 2016 until 30th June 2023 were used. The local Medical Research Ethics Committee approved the studies (Approval Code NL55755.018.15 and NL61882.048.17), and participants signed informed consent forms prior to commencing the study. The study was conducted in accordance with the Declaration of Helsinki and the Medical Research Involving Human Subjects Act (WMO).

### Measures

A medical professional assessed patients’ height and demographic data (i.e., age, sex, and race) during the preoperative assessment, and weight at each hospital visit. Patients completed an online self-report survey including questions about their education, occupation, marital status, and psychological questionnaires at each assessment point. Weight was reported as BMI (kg/m^2^) and weight loss in percent of adjustable weight loss (%AWL) and percentage of total weight loss (%TWL). Psychological variables were measured with Dutch versions of psychometrically validated scales when available.

#### Psychological health

*HR-QoL.* The Impact of Weight on Quality of Life (IWQOL-Lite [46, 47]) scale is a reliable and valid questionnaire developed specifically for measuring weight-related QoL. It is comprised of 31 items measuring five aspects of HR-QoL (i.e., physical functioning, self-esteem, sexual life, public distress, work life), answered on a 5-point Likert scale. The total scores range from 0 to 100, with higher scores denoting better HR-QoL. The internal reliability in the current sample was (Cronbach’s alpha, *α*) 0.93.

*Depression*. The Center for Epidemiology Studies Depression Scale (CES-D [48, 49]) is a 20-item questionnaire used to measure depressive symptoms, answered on a 4-point scale (*α* = 0.85). The total score ranges from 0 to 60, with higher score denoting more severe depression.

#### Psychological measures preoperative

Furthermore, we assessed patients’ psychological characteristics before their BMS. We prioritised disease-specific instruments whenever possible to accurately capture the unique impacts of obesity on wellbeing. *The Body Image Scale* [50] was used to measure patients’ perceptions of and attitudes toward the appearance of their own body (the tenth item regarding surgery scar was omitted, thus nine items score range 0–27, α = 0.82). *Weight Efficacy Lifestyle Questionnaire* [51] measured individuals’ confidence in their ability to control their eating behaviours in different situations (20 items, score range 0–100, α = 0.96). *Food Craving Questionnaire-Trait* [52] was used assess the frequency and intensity of food craving experiences (21 items, score range 0–100, α = 0.95). Patients confidence to plan and execute physical activities and exercise based on their own volition was measured with the *SCI Exercise Self-Efficacy scale* [53] (10 items, scale range 10–40, α = 0.90). The total time in minutes spent exercising during the past week was measured with the *Exercise behaviour scale* [54] (six items, scale range 0–1080, α = 0.35). Patients’ neuroticism, i.e., tendency toward emotional instability and negative feelings, and conscientiousness, i.e., inclination to be responsible, organized and careful, were measured with the *NEO-FFI-R scale* [55], (12 items for each subscale, scale range 12–60, α = 0.84 and 0.84, respectively). *Self-compassion scale Short Form* [56] was used to assess patients’ ability to treat their feelings or suffering with a sense of kindness, connection and mindfulness (12 items, scale range 12–84, α = 0.75). *Rosenberg Self-esteem scale* [57] was used to measure individual’s overall sense of personal worth and abilities (10 items, scale range 0–30, α = 0.87). The characteristic way of relating to others in intimate relationships, i.e., attachment style, was measured with the *Experience in Close Relationships Scale* [58] anxiety subscale (eight items, scale range 8–64, α = 0.85), and avoidance subscale (eight items, range 8–64, α = 0.75). *De Jong-Gierveld Loneliness Scale* [59] was used to assess the feelings of missing an intimate relationship and belonging (11 items, scale range 0–11, α = 0.92). The extent of feeling supported by close friends or family, both in generally positive and negative way, as well as in relation to eating and exercising habits, were measured using *the Social Support List* [60, 61], (six items for positive and negative subscale each, scale range 6–30, α = 0.91 and α = 0.72, respectively), and *Social Support for Dieting and Exercise scales* [62] (10 items for diet subscale, scale range 10–50, α = 0.64, and 13 items for exercise subscale, scale range 13–65, α = 0.89).

### Statistical analysis

We identified subgroups of patients with different trajectories over time for BMI, HR-QoL and depression using Latent Class Growth Analysis (LCGA) [63, 64]. LCGA is used to explore patterns of development in data over time and probabilistically assign individuals into subgroups based on their common pattern of development [63–65]. LCGA aims to identify latent or unobserved classes based upon similar growth characteristics, and estimating an individual’s probability of belonging in a certain class [63, 64]. Time was treated as a categorical variable, thus, piecewise per wave, without imposing any a priori assumption about the shapes of the time trends. Residual variances were allowed to vary over time and across classes, as this method leads to better class recovery, classification accuracy and less bias [66]. Models with increasing number of trajectories (classes) were estimated. The final models were selected based on (a) the lowest Akaike (AIC) and Bayesian information criterion (BIC) indexes, with lower values indicating better fit [67]; (b) not less than 5% of participants in a class; and (c) adequate clinical interpretability. The LCGA models were estimated in Mplus version 8.7 [68] with 2500 starting values and 150 final iterations. Models were estimated using full information maximum likelihood (FIML) and robust standard errors on all available data for better handling of missing data, assuming missing to be at random.

#### Associations between trajectories

To evaluate the associations between the trajectories of BMI, HR-QoL, and depression, we fitted two additional models in which the trajectory membership of HR-QoL and depression was predicted by the trajectory membership of BMI. The trajectory parameters were fixed for BMI, HR-QoL, and depression based on the final model chosen in the LCGA: only the cross-classification membership probabilities from BMI trajectory membership to HR-QoL and depression trajectory memberships were estimated. Lastly, we tested whether the distribution of membership probabilities of BMI trajectory classes differed across all HR-QoL and depression classes.

#### Associations between preoperative characteristics and trajectories

Preoperative psychological characteristics and demographic factors were analysed as descriptors of the outcome trajectories using means and 95% confidence intervals of the continuous measures within each class. For categorical variables, we estimated the frequencies of distribution in percentages per each class. Marital status was categorised as either ‘married/partnered’ or ‘other’ (including single, widowed, and divorced). Educational level was grouped into ‘high school or lower’ (including none, lower general education, and high school) and ‘vocational/higher education’ (including secondary vocational, higher professional, and university education). Employment status was classified into ‘full-/part-time employment’ and ‘no work/other’ (including homework, disabled, volunteer work, searching for work, studying, and retired). In order to prevent bias due to classification error, these descriptive analyses were estimated weighting by each class membership probability. By comparing the confidence intervals across classes, we explored which continuous baseline characteristics differed across the trajectory classes.

## Results

### Sample characteristics

**Fig. 1:**
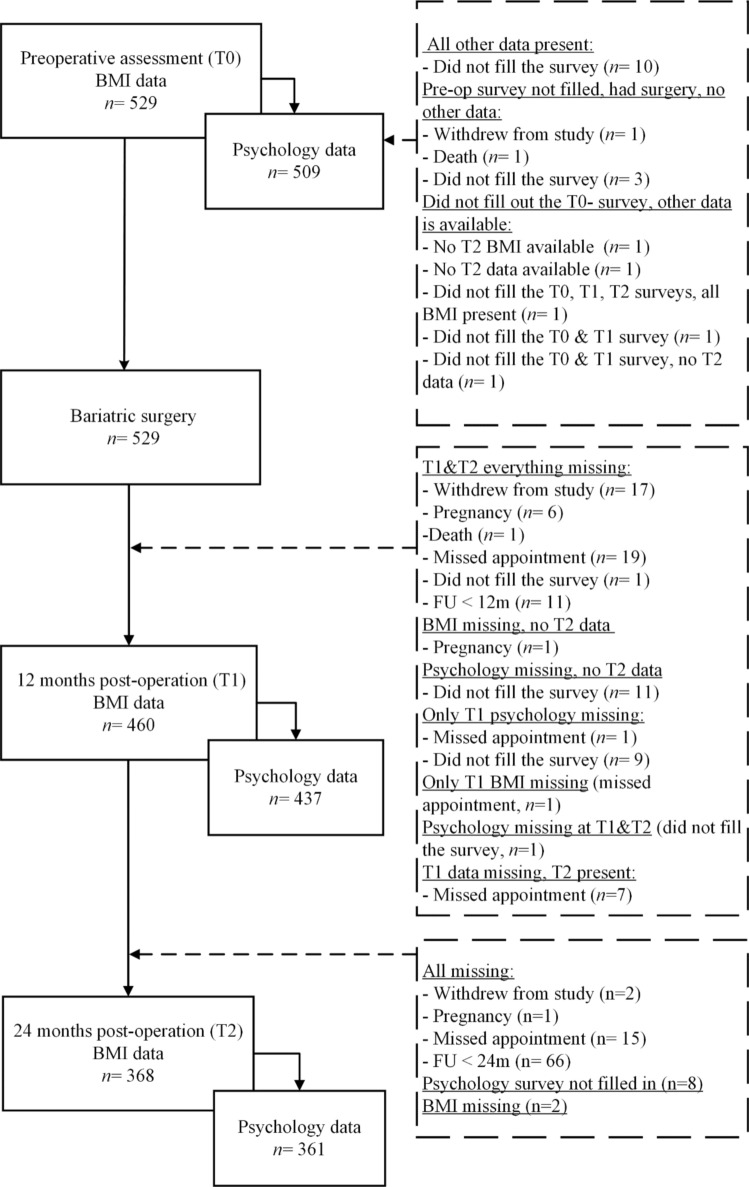
Flowchart of participant inclusion

A total of 598 participants were included in the study, of which 529 (88.46%) participants had undergone BMS. Patients who had not undergone the surgery (*n* = 69) were excluded from the current analyses. This resulted in weight data being available from 529 patients at pre-operation, and 460 patients and 368 patients at 12 and 24 months post-operation, respectively. Similarly, preoperative psychology data was available from 509 patients, and 437 and 361 patients from the follow-up assessments. Accounting for at least one non-missing data point in the three waves, weight data was available from 529 patients and psychology data from 522 patients. See Fig. 1 for the inclusion of data.

Of the 529 patients 408 (77.1%) were female. Patients were on average 47 (SD = 11) years old, and had a preoperative BMI of 39.13 (SD = 3.96) kg/m^2^. The majority were married or had a partner (71.1%), and identified as Caucasian (85.8%) as reported in Table 1. The Roux-en-Y gastric bypass was performed on 451 patients (85.3%), laparoscopic omega loop gastric bypass on 52 patients (9.8%), and sleeve gastrectomy on the remaining 26 patients (4.9%). Patients included in the analyses (*n* = 329) were, on average, 2.2 years older (SE of difference = 0.9 years, *p* < 0.05) and had a lower preoperative BMI (difference = 1.0 ± 0.3 kg/m^2^, *p* < 0.05) compared to those excluded (*n* = 269), with no other significant differences observed between the groups.

**Table 1:** Sociodemographic characteristics of participants at baseline (n = 529)

	*n*	%
Gender, female	408	77.10
Age (mean, SD) [range 19.1–66.1 years]	46.69	10.84
*Race*		
Caucasian	454	85.8
South American	23	4.3
North African	12	2.3
Mediterranean	11	2.1
Black	10	1.9
East Asian	6	1.1
West Asian	6	1.1
Other	4	0.8
Middle Asiatic	2	0.4
Slavic	1	0.2
Marital status*^22^		
Married/partnered	376	71.1
Single	94	17.8
Divorced/widowed	37	7.0
Relationship duration in years (mean, SD)*^18^	13.78	13.29
*Having children, yes (median* = 2 children)*^18^	396	74.9
Highest educational level *^22^		
No	1	0.2
Lower general education/primary education, or a part of it	10	1.9
General education/high school (lbo, vbo, mavo, mulo, ulo, havo, vwo, hbs, gymnasium)	148	28.0
Secondary vocational education (mbo, mts)	225	42.5
Higher professional education (hbo, hts)	98	18.5
Scientific education (university)	25	4.7
Employment*^21^		
Employed (full or part time)	396	74.9
Homework	32	6
Disabled for work	31	5.9
Voluntary/unpaid work	20	3.8
Searching for work	17	3.2
Study	8	1.5
Retired	4	0.8
Start of obesity *^8^		
Childhood	168	31.8
Puberty	131	24.8
Adulthood	222	42.0
*Type of surgery*		
Roux-en-Y Gastric Bypass	451	85.3
Laparoscopic Omega loop Gastric Bypass	52	9.8
Sleeve gastrectomy	26	4.9

*^XX^ = Data missing from XX participants (e.g., *^21^ = Data missing from 21 participants)

### Development of BMI

**Fig. 2:**
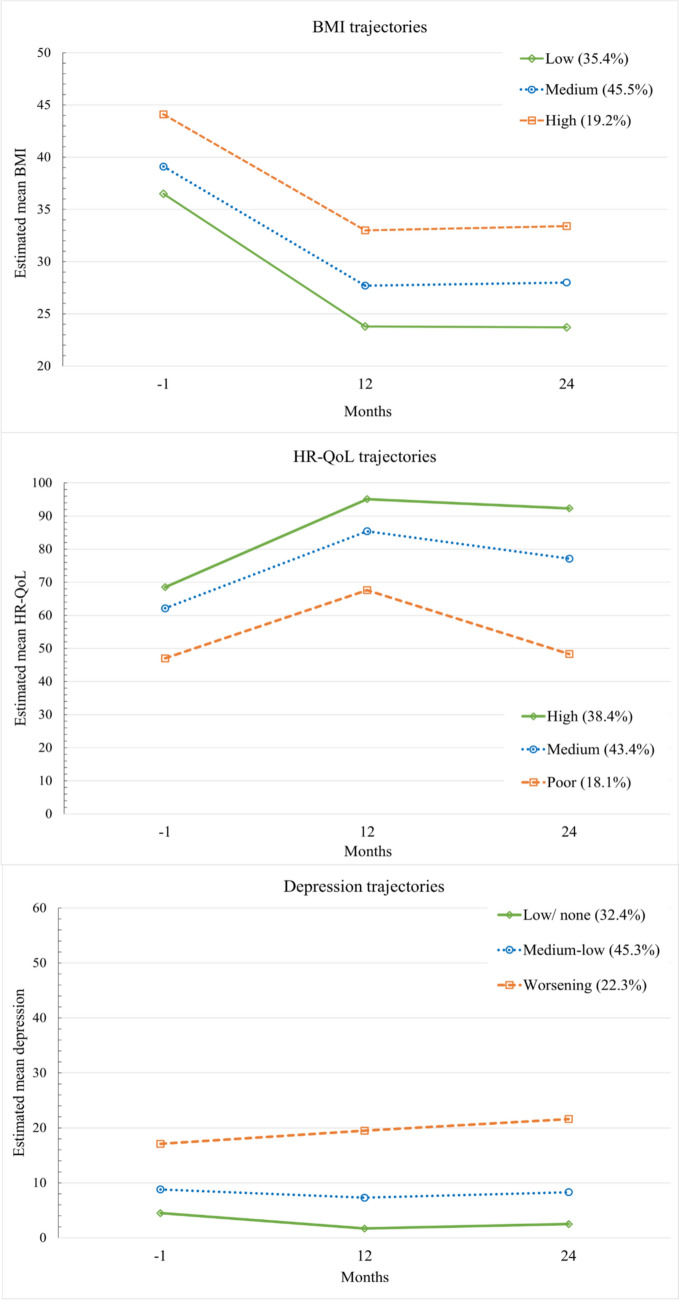
Longitudinal trajectories of BMI, HR-QoL, and depression from pre-operation (month − 1) to 24 months post-operation

As expected, the decrease of weight on the total group level was significant and varied in its extent across patients (Table 2 and Fig. 2). The model with four classes had slightly better fit statistics than the three-class model (see Table 3), but as the fourth class did not present as clinically different from the three classes, a model with three trajectories was chosen. Trajectory 1 (35.4% of patients), labelled as the “Low” trajectory, started with the lowest BMI (36.5 kg/m^2^), lost the most weight at 12-month follow-up (− 12.7 kg/m^2^), which they kept stable until the 24-month follow-up, ending with a TWL of 35.1% and AWL of 54.5%. The second trajectory, labelled “Medium”, representing 45.5% of patients, started with a BMI of 39.1 kg/m^2^, and achieved substantial weight loss by the 12-month follow-up (29.2%TWL or 43.7%AWL). Weight loss plateaued thereafter, resulting in an average of 11 kg/m^2^ loss, and 28.4% TWL or 42.5% AWL by the 24-postoperative month. Trajectory 3 (19.2% of patients), denoted the “High” trajectory, commenced with the highest preoperative BMI at 44.1 kg/m^2^. By the 12-month follow up, patients lost − 11.1 kg/m^2^, and by the 24 postoperative month reached an average weight loss of 10.7 kg/m^2^ (24.3% TWL or 34.4% AWL). Overall BMI improved, but some individuals regained some of their weight back 24 months after the surgery (see Online Appendix Figure A1).

**Table 2:** Mean values, residual variance, and the mean differences over time for the total sample and for the distinct trajectories of BMI, HR-QoL, and depression

	T0	T1	T2	T1–T0	T2–T1	T2–T0
	Mean (residual variance)			Mean difference		
BMI						
Total sample	39.1 (15.7)	27.3 (14.8)	27.6 (16.7)	− 11.8*	0.3*	− 11.5*
*Distinct trajectories*						
Low (35.4%)	36.5 (5.5)	23.8 (2.6)	23.7 (3.6)	− 12.7*	− 0.1	− 12.8*
Medium (45.5%)	39.1 (7.2)	27.7 (2.1)	28.0 (2.9)	− 11.3*	0.3*	− 11.0*
High (19.2%)	44.1 (17.3)	33.0 (10.3)	33.4 (11.1)	− 11.1*	0.4	− 10.7*
HR-QoL						
Total sample	61.8 (297.7)	86.0 (156.3)	77.6 (388.5)	24.2*	− 8.4*	15.8*
*Distinct trajectories*						
High (38.4%)	68.5 (286.9)	95.1 (6.8)	92.3 (21.6)	26.6*	− 2.8*	23.8*
Medium (43.4%)	62.1 (194.3)	85.4 (46.2)	77.1 (123.9)	23.3*	− 8.3*	15.0*
Poor (18.1%)	47.0 (257.6)	67.6 (234.2)	48.3 (455.0)	20.6*	− 19.3*	1.3
Depression						
Total sample	9.2 (52.6)	8.0 (68.8)	9.3 (86.5)	− 1.2*	1.3*	0.1
*Distinct trajectories*						
Low/none (32.4%)	4.5 (12.6)	1.7 (1.9)	2.5 (4.8)	− 2.7*	0.8*	− 1.9*
Medium–low (45.3%)	8.8 (22.1)	7.3 (12.8)	8.3 (21.9)	− 1.4*	1.0	− 0.4
Worsening (22.3%)	17.1 (76.7)	19.5 (102.7)	21.6 (128.3)	2.4	2.1	4.4*

T0 = preoperative assessment, T1 = 12 months postoperative assessment, T2 = 24 months postoperative assessment

* *p* < .05

**Table 3:** Fit statistics for trajectories of BMI, HR-QoL, and depression

Number of trajectories	AIC	BIC	aBIC	Entropy	Npar	Smallest class size (%)
*Trajectories of BMI*						
1	7594.1	7619.7	7600.7	−	6	100.0
2	7103.4	7158.9	7117.6	0.763	13	28.6
3	6846.3	6931.7	6868.2	0.761	20	19.2
4	6715.2	6830.5	6744.8	0.790	27	7.7
5	6657.7	6802.9	6695.0	0.801	34	7.7
6	6618.9	6794.0	6663.8	0.804	41	4.8
7	6588.1	6793.1	6640.7	0.818	48	2.8
*Trajectories of HR-QoL*						
1	10,980.6	11,006.1	10,987.1	−	6	100.0
2	10,451.2	10,506.5	10,465.3	0.664	13	49.1
3	10,291.4	10,376.6	10,313.1	0.658	20	18.1
4	10,261.3	10,376.3	10,290.6	0.607	27	13.0
5	10,244.3	10,389.0	10,281.1	0.664	34	0.8
*Trajectories of depression*						
1	9204.4	9229.9	9210.9	−	6	100.0
2	8455.0	8510.4	8469.1	0.758	13	36.9
3	8262.8	8347.9	8284.5	0.718	20	22.3
4	8191.4	8306.4	8220.7	0.700	27	11.7
5	8161.7	8306.5	8198.6	0.674	34	6.0
6	8137.4	8312.0	8181.9	0.681	41	5.4
7	8127.0	8331.4	8179.0	0.690	48	5.2

* Npar * number of parameters

Further, 24 months after the surgery, 72.4% patients in the Low trajectory (thus, 89 out of the 368 participants at 24 months), 1.7% (*n* = 3) patients in the Medium trajectory, and none of the High trajectory patients reached a healthy weight of BMI < 25 kg/m^2^. All patients in the Low and Medium trajectories, and 79.5% of patients (*n* = 58) in the High trajectory reached a BMI below 35 kg/m^2^. Conversely, 20.6% of the High trajectory patients (*n* = 15) had a BMI above 35 kg/m^2^ (thus, 4.1% of total sample).

### Development of HR-QoL

On the total sample level, HR-QoL improved over time, but differed across patients. A HR-QoL model with three trajectories was chosen based on the fit statistics and interpretability of the emerging trajectories. The fourth trajectory in the four-class model did not present clinically relevant differences to the three-class solution. Patients in the first trajectory, the “High” trajectory (38.4% of patients), started with the highest preoperative HR-QoL, improved the greatest, and remained stable by the end of study period. The second trajectory (43.4%), labelled as the “Medium”, showed first a great improvement in HR-QoL, followed by a partial decline by the 24th postoperative month. The “Poor” trajectory (18.1%) was characterized by the poorest initial HR-QoL values, which improved significantly by the 12-month follow-up but subsequently regressed to the preoperative levels by the 24th postoperative month.

### Development of depression

While depression remained relatively stable across the entire study cohort, subtle variations were observed across patients. Again, the model with four classes had lower BIC and AIC values, but the fourth class solution did not add a clinically different trajectory compared to the three-class model. Thus, a model with three trajectories was selected. The first, “Low/none”, trajectory included 32.4% of patients, who were characterized by initially low depressive scores, followed by minor improvement and subsequent stability until the 24-month postoperative assessment. The largest trajectory comprised of 45.3% of patients and was labelled “Medium–low”. This trajectory was characterised by a consistent low level of depressive symptoms throughout the study period. The third trajectory, labelled the “Worsening” trajectory, comprised of 22.3% of patients, displayed a gradual exacerbation of depression over the study period.

### Associations between trajectories of BMI and trajectories of HR-QoL

The association between the BMI and HR-QoL trajectory classes appeared to be indistinct (Table 4). The distribution of the class membership probabilities of HR-QoL trajectories in BMI trajectories did not differ from the expected probabilities under independency (i.e., the class membership distribution of HR-QoL trajectories was similar across all the BMI trajectory classes). Further, the test for the equality of probabilities was not significant, implying that the BMI trajectories were equally distributed across all HR-QoL trajectories (*p* = 0.434).

**Table 4:** Association between BMI and HR-QoL and depression trajectory classes membership

Class membership probability distribution of HQOL and Depression trajectories for each BMI trajectory class (row percentage)								
	Overall HR-QoL				Overall depression			
	High (%)	Medium (%)	Poor (%)		Low/none (%)	Medium–low (%)	Worsening (%)	Overall BMI (%)
Trajectories of BMI				Trajectories of BMI				
Low	34.3	47.1	18.5	Low	26.2	43.1	30.8	35.4
Medium	43.8	40.7	15.5	Medium	38.8	45.0	16.2	45.5
High	32.9	43.3	23.8	High	28.5	49.9	21.7	19.2
Overall HR-QoL	38.4	43.5	18.2	Overall Depression	32.4	45.2	22.4	

### Associations between trajectories of BMI and trajectories of depression

The prediction that trajectories of BMI and depression development would match was partially supported by our data (Table 4). Individuals within the Low BMI trajectory were slightly less likely to belong in the Low/none depression trajectory class (26.2% observed distribution versus 32.4% expected distribution under independence), and more likely to belong in the Worsening depression class (30.8%) than expected under independency (22.4%). Patients belonging to the Medium BMI trajectory class were less likely to belong to the Worsening depression trajectory class and slightly more likely to belong to the Low/none depression trajectory class than expected under independency. The Wald-test for equality of probabilities was bordering significance, implying that the BMI trajectories were not equally distributed across the trajectories of depression (*p* = 0.046).

### Psychological profiles

The mean and 95% confidence intervals for the psychological characteristics before operation for each trajectory of BMI, HR-QoL, and depression are presented in Table 5. Patients within the Low BMI trajectory were less satisfied with their body image than patients in the Medium and High trajectories, and had lower self-esteem than patients in the Medium trajectory. No other differences were found between the BMI trajectories. Patients who started with the poorest HR-QoL (Poor-trajectory) scored also the poorest on most psychological characteristics compared to patients within the two other trajectories. Specifically, they had the lowest body image satisfaction, lowest self-efficacy to exercise, lowest conscientiousness, highest neuroticism, poorest self-compassion and self-esteem, highest attachment avoidance and anxiety, highest loneliness and highest negative social support. Conversely, patients within the High HR-QoL-trajectory scored the highest on the aforementioned characteristics, and the scores of Medium patients were in between these classes. Patients within the Worsening depressive symptom trajectory had the poorest preoperative psychological scores compared to the two other trajectories. Patients within the Worsening trajectory reported poorest body image satisfaction, self-efficacy to control their eating behaviours and self-efficacy to exercise, experienced most food cravings, and had the poorest psychological profile with high neuroticism, low consciousness, poor self-esteem and self-compassion, high attachment avoidance and anxiety, loneliness and reporting the poorest social support. Patients within the Low/none depression trajectory had a slightly more ‘positive’ preoperative personality profile compared to the constant low trajectory. There was no age effect, i.e., there were no differences in average age between the different trajectories for BMI, HR-QoL, or depression. The results suggest that more females than average may be present in the Low BMI trajectory, as well as in the Poor HR-QoL and Worsening depression trajectories. In the Poor HR-QoL trajectory group, fewer patients were married or partnered and employed either full- or part-time. Conversely, in the Low depression trajectory group, a higher proportion of patients were married or partnered and employed full- or part-time. Education level appeared similar across trajectories.

**Table 5:** Patients’ characteristics before operation for different trajectories of BMI, HR-QoL, and depression

	BMI trajectories			HR-QoL trajectories			Depression trajectories		
	Low (*n* = 187)	Medium (*n* = 241)	High (*n* = 101)	High (*n* = 196)	Medium (*n* = 221)	Poor (*n* = 92)	Low/none (*n* = 165)	Medium- low (*n* = 231)	Worsening (*n* = 113)
Body image satisfaction	10.4 (9.9–11.0)	12.1 (11.6–12.5)	12.6 (11.8–13.3)	12.6 (12.1–13.2)	11.5 (11.1–11.9)	9.6 (8.8–10.3)	12.5 (11.9–13.1)	11.7 (11.3–12.1)	10.0 (9.3–10.7)
Self-efficacy eating	66.6 (64.3–69.0)	67.1 (64.9–69.3)	66.3 (62.8–69.8)	69.7 (67.3–72.1)	65.3 (63.4–67.3)	64.0 (60.9–67.1)	69.0 (66.4–71.7)	67.3 (65.2–69.4)	62.5 (59.5–65.4)
Food cravings	44.9 (42.2–47.6)	43.7 (41.3–46.1)	43.5 (39.7–47.3)	41.2 (38.5–43.9)	44.8 (42.7–46.8)	48.8 (45.2–52.5)	42.3 (39.4–45.2)	43.0 (40.7–45.2)	49.1 (45.6–52.5)
Self-efficacy exercise	31.9 (31.1–32.7)	32.5 (31.8–33.2)	32.7 (31.7–33.6)	33.4 (32.7–34.1)	32.2 (31.6–32.8)	30.5 (29.4–31.5)	33.4 (32.7–34.1)	32.5 (31.8–33.1)	30.4 (29.4–31.4)
Physical activity	169.5 (151.9–187.1)	164.9 (148.6–181.1)	143.7 (122.9–164.6)	169.2 (152.2–186.2)	159.9 (145.1–174.6)	154.4 (131.7–177.1)	169.1 (150.5–187.6)	168.6 (152.6–184.6)	140.6 (121.4–159.9)
Neuroticism	30.3 (29.2–31.4)	28.5 (27.5–29.6)	28.9 (27.4–30.4)	26.1 (25–27.1)	29.6 (28.8–30.4)	35.2 (33.9–36.5)	24.7 (23.7–25.6)	29.2 (28.3–30)	36.1 (34.9–37.3)
Conscientiousness	47.1 (46.3–48.0)	47.3 (46.6–48.1)	46.3 (44.9–47.6)	48.3 (47.4–49.2)	46.9 (46.2–47.6)	44.9 (43.8–46.0)	48.7 (47.9–49.6)	46.9 (46.2–47.7)	44.9 (43.7–46.0)
Self-compassion	55.8 (54.0–57.6)	58.4 (56.8–60)	57.6 (55.5–59.7)	61.5 (59.9–63.2)	56.8 (55.4–58.2)	49.6 (47.4–51.8)	63.5 (61.8–65.2)	57.2 (55.8–58.5)	48.6 (46.5–50.7)
Self-esteem	19.3 (18.6–20.0)	21.0 (20.4–21.6)	20.0 (19.1–21)	22.0 (21.3–22.6)	20.1 (19.6–20.6)	16.8 (16.0–17.6)	22.5 (21.9–23.1)	20.4 (19.8–20.9)	6.6 (15.8–17.5)
Attach. avoidance	21.3 (20.2–22.5)	21.0 (20.0–22.0)	20.4 (19.0–21.8)	19.0 (18.0–20.0)	20.9 (20–21.8)	25.5 (24.0–26.9)	19.1 (18.0–20.2)	20.6 (19.7–21.5)	24.6 (23.1–26.1)
Attach. anxiety	23.5 (22.0–25.0)	22.5 (21.3–23.7)	23.2 (21.1–25.3)	19.4 (18.2–20.5)	23.3 (22.2–24.4)	29.9 (28.0–31.9)	18.6 (17.4–19.8)	23.0 (21.9–24.1)	29.4 (27.4–31.3)
Loneliness	2.2 (1.8–2.6)	2.0 (1.7–2.3)	1.5 (1.1–1.9)	1.3 (1.0–1.6)	1.9 (1.6–2.2)	3.6 (3.0–4.2)	0.8 (0.6–1.1)	1.9 (1.6–2.2)	3.8 (3.2–4.4)
Pos social support	18.2 (17.7–18.7)	18.2 (17.8–18.7)	17.7 (17.2–18.2)	18.7 (18.3–19.2)	17.9 (17.5–18.3)	17.5 (16.9–18.1)	18.9 (18.4–19.4)	18.1 (17.7–18.4)	17.1 (16.5–17.7)
Neg social support	8.6 (8.2–8.9)	8.4 (8.1–8.7)	8.4 (8.0–8.8)	7.9 (7.7–8.2)	8.6 (8.3–8.8)	9.4 (8.9–9.8)	7.7 (7.5–8.0)	8.4 (8.1–8.6)	9.7 (9.2–10.1)
Support eating	34.9 (34.3–35.6)	34.4 (33.8–35)	34.4 (33.7–35.2)	35.0 (34.4–35.7)	34.3 (33.8–34.9)	34.3 (33.5–35.1)	34.8 (34.1–35.6)	34.9 (34.4–35.4)	33.7 (32.8–34.6)
Support exercise	31.1 (29.9–32.3)	31.2 (30.1–32.2)	30.5 (28.9–32)	31.7 (30.5–32.8)	30.9 (29.9–31.9)	29.9 (28.6–31.2)	31.6 (30.3–32.9)	30.8 (29.8–31.8)	30.6 (29.3–32.0)
Age	47.0 (45.6–48.3)	46.7 (45.4–48.0)	46.2 (44.1- 48.2)	46.5 (45.2–47.9)	47.2 (46.0–48.4)	46.0 (44.0–48.1)	46.6 (45.1–48.0)	46.6 (45.4–47.9)	47.1 (45.3–48.8)
*Gender*									
Male	16.2	26.2	27.3	26.9	22.9	14.1	26.0	23.8	16.3
Female	83.8	73.8	72.7	73.1	77.1	85.9	74.0	76.2	83.7
*Marital status*									
Married/partnered	73.6	76.2	69.6	78.1	74.6	64	81.2	71.1	69.3
Other	26.4	23.8	30.4	21.9	25.4	36	18.8	28.9	30.7
*Education*									
High school or lower education	31.2	29.4	30.5	29.2	30.8	33.3	31.9	27.9	34.4
Vocational/higher education	68.8	70.6	69.5	70.8	69.2	66.7	68.1	72.1	65.6
*Employment*									
Full-/part-time	76.4	80.7	74.2	81.6	81.8	61	86.3	80.1	61.6
No work/other	23.6	19.3	25.8	18.4	18.2	39	13.7	19.9	38.4

Data presented as means, 95% confidence interval in brackets, except categorical variables presented as within-class %

## Discussion

This study sought to investigate the concurrent development of weight, HR-QoL, and depression after BMS, along with their interrelationships and preoperative traits characterizing them. As expected, most patients lost a significant amount of weight and their psychological health improved in the 24 months following bariatric metabolic surgery, yet distinct patterns of change in BMI, HR-QoL and depression were observed. In contrast to our expectations, there was no relation between the BMI and HR-QoL trajectories. However, a weak association between the BMI trajectory classes and depression trajectory classes was found. Lastly, the three BMI trajectories had comparatively similar preoperative psychological profiles, but distinctions were found for the trajectories of HR-QoL and depression.

Three trajectories of BMI were distinguished, labelled as a Low, Medium, and High trajectory, mainly characterized by varying preoperative BMIs. This finding aligns with prior research, exhibiting similar patterns but varying in the extent of total adjustable weight loss [5, 6, 13–17]. The differing number of identified trajectories in the literature may be attributed to variations in sample sizes, characteristics, and follow-up periods across earlier studies. The majority of patients in our study demonstrated a favourable pattern in weight loss, showing apparent sustained reductions. Among the entire sample, 25% achieved a healthy BMI of less than 25 kg/m^2^ 24 months after the operation, while nearly all patients (96%) attained a BMI below 35 kg/m^2^. In contrast, a minority (4%) exceeded the BMI of 35 kg/m^2^, implying a poor surgical outcome or weight regain between the 12 and 24 postoperative months. This aligns with prior findings indicating that weight regain typically starts 2–5 years post-BMS [3–6, 69, 70]. Patients starting with lower preoperative BMIs achieved the greatest weight loss, with over 70% reaching a healthy BMI. Higher preoperative BMI typically correlates with greater weight loss after surgery [5, 71]. However, in line with our results, some studies have also indicated that higher preoperative weight is linked to inferior weight loss outcomes [72, 73]. This highlights the complexity of predicting weight loss outcomes from patients’ preoperative weight, and underscores the importance of looking at patients' health from a wider point of view.

Patients in the Medium BMI trajectory were more likely to belong to the Low/none depression trajectory class, who also had a better preoperative psychological profile compared to the other two depression trajectories. Thus, the Medium BMI trajectory patients reached a satisfactory weight loss result, and were most likely to be better off in terms of their overall psychological condition. Conversely, most patients in the Low BMI trajectory reached a healthy BMI, but unexpectedly, were more likely to follow the Worsening depression trajectory. The Low BMI trajectory patients reported lower satisfaction with body image and poorer self-esteem before operation compared to counterparts in the other two trajectories. This outcome is intriguing as these patients achieved the most favourable weight loss result, yet seemed to struggle the most with their psychological health (i.e., depression and poorer preoperative psychological profile). It is perhaps important to keep in mind that patients in the Low BMI trajectory had an average BMI below 40 kg/m^2^, and thus had to have a comorbid medical condition related to obesity in order to receive a BMS. Thus, patients in the Low trajectory probably suffered from multimorbidity, possibly explaining their higher dissatisfaction with their health. These findings also support the hypothesis that patients with poorer self-esteem may experience greater distress from overweight, and are therefore highly motivated to seek surgical treatment and adhere to medical and dietary guidelines [74]. Patients on the lower spectrum of obesity may pursue surgical help for reasons such as improving their body image and self-worth, rather than solely aiming to alleviate obesity-related medical conditions. Improvement of HR-QoL serves as a significant motivator for seeking surgery [23], and has been argued, together with resolution of comorbidities, to be more relevant surgical outcome than weight loss above a certain threshold [75].

The relationship between depression and weight loss outcomes has been a much debated question. One perspective suggests that the majority of patients are depressed due to their weight and its associated limitations. Consequently, actively engaging in lifestyle changes through surgery may lead to a reduction in depression. An opposing viewpoint posits that higher levels of psychopathology hinder patients' ability to adhere to treatment and lifestyle recommendations, potentially exacerbating surgical outcomes [76, 77]. While depression is usually regarded as a predictor of poor weight loss [77], conflicting findings have indicated the opposite [78, 79]. Thus, patients with poorer mental health status may be more motivated to lose weight and improve their psychological wellbeing, but may continue to struggle with their mental health despite achieving great weight loss results. This finding underscores the complicated relationship between obesity and depression, as well as the importance of monitoring patients’ psychological wellbeing throughout the treatment. Clinicians should be aware of this complex relationship and incorporate evaluation of psychological wellbeing in the long-term follow-up after the operation. Continuous psychological support and improved methods for detecting mental health issues are essential, along with comprehensive postoperative care that addresses both physical and psychological needs.

Regarding the psychological outcomes of this investigation, HR-QoL and depression improved for most patients after the surgery, though distinct trajectories were identified. While patients initially improved in their HR-QoL, a subgroup of patients (~ 20%, Poor trajectory) regressed to their poor preoperative level of HR-QoL, leaving them less satisfied with their wellbeing 24 months postoperatively than they were before the surgery. They also had the poorest preoperative psychological profile compared to the other HR-QoL trajectories. This finding is consistent with the existing literature describing different numbers of HR-QoL trajectories, highlighting the variability in psychological outcomes [17, 24]. Although patients lost a significant amount of weight and their HR-QoL improved in the 24 months following the surgery, we did not find a significant association between the trajectories of HR-QoL and BMI, meaning that in our study, the probability of belonging to one of the HR-QoL trajectory classes was independent of the patients’ membership of BMI trajectory class. Earlier studies have found changes in BMI to match the changes in HR-QoL [24–26]. However, our study shows that following a favourable BMI trajectory does not necessarily result into a favourable psychological health trajectory. The lack of a clear relationship between the BMI and HR-QoL trajectories in our study may stem from BMI trajectories primarily being defined by initial weight, while HR-QoL trajectories varied more in their patterns.

A novel finding was the identification of three distinct trajectories of depression. The majority of patients exhibited minimal depressive symptoms that remained consistently low throughout the study period. However, a considerable group of patients (22.3%) experienced a deterioration in depressive symptoms. Patients within the Worsening trajectory scored the poorest on all psychological measures, especially exhibiting poor self-esteem and self-compassion and high attachment anxiety compared to the other two depression trajectories. These findings suggest that most patients undergoing BMS do not suffer from depression, however, a subgroup of patients may be at risk of deteriorating depressive symptoms. It may be that these patients are more susceptible to psychological difficulties, and vulnerable during negative life events and experiences, which could lead to further complications in their psychological health.

**Fig. 3:**
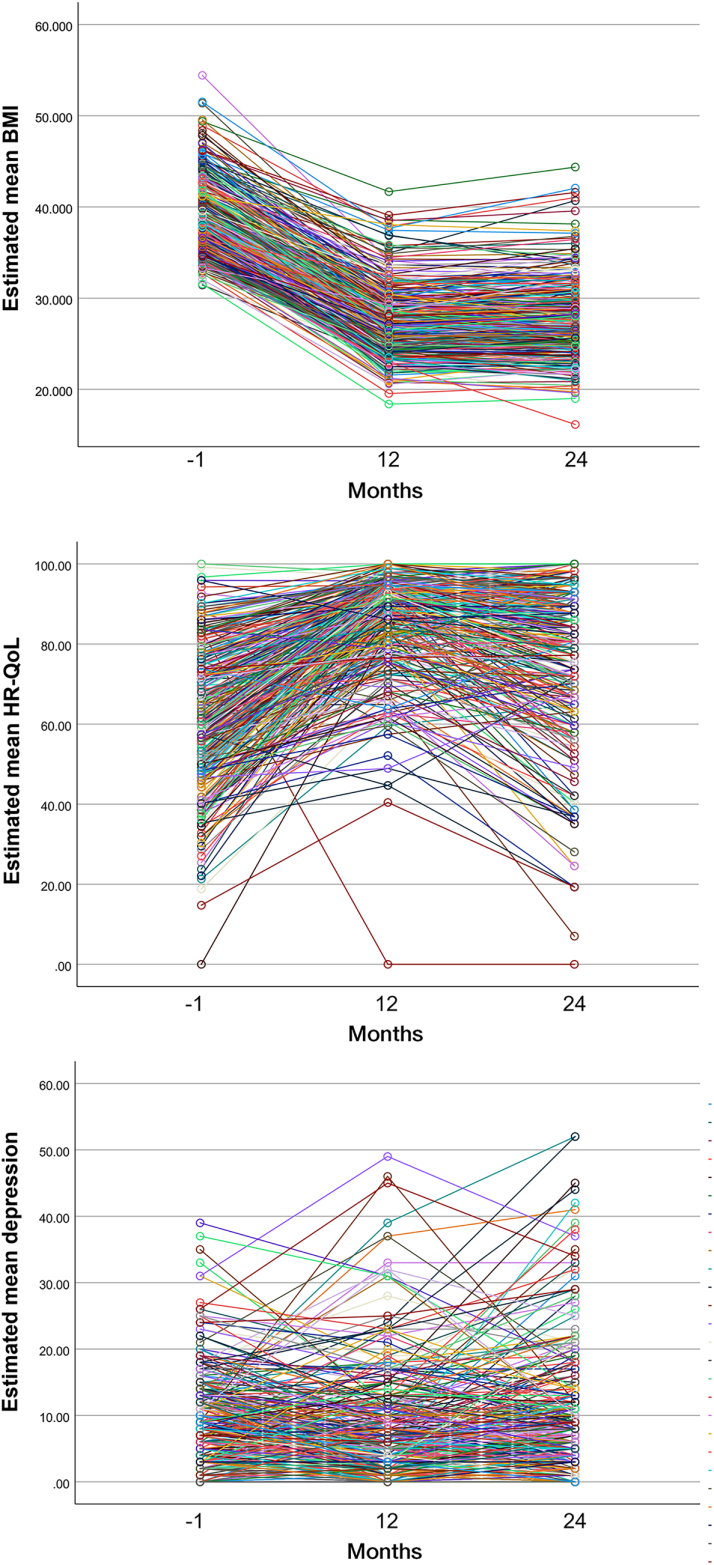
Spaghetti plots displaying the estimated mean of the outcome variables (BMI, HR-QoL, and depression) from month − 1 to 24 months after BMS

It is worth noting that while our analyses yielded these three trajectories, these classes do not have to represent the only (well-separated) subclasses of patients, but rather reflect a summary (or categorization) of trends within a broader and heterogeneous and continuous spectrum of BMI, HR-QoL and depression trajectories. This continuous spectrum in the three outcome variables is well-depicted in the spaghetti plots in Fig. 3.

### Strengths and limitations

Strengths of this study include the longitudinal design, the large sample size of patients with obesity, assessment of numerous psychological characteristics and health outcomes and inclusion of different surgical techniques. Further, patients were assessed after they had been accepted for surgery, thus patients could answer the psychological survey honestly without worrying portraying themselves in a manner that could be considered as a contraindication for BMS. This study’s limitations include the recruitment of patients from two Dutch hospitals, predominantly comprising individuals of Caucasian ethnicity and female gender. Consequently, the generalizability of the findings may be constrained to more diverse populations, encompassing variations in nationality, cultural context, internationality, race, and gender. Lastly, a longer follow up period is needed to investigate whether these trajectories and their relations sustain over time. Previous studies have shown that improvements in HR-QoL can be sustained for up to 12 years after BMS [25], and decreases in depression symptoms 3 years postoperatively [21]. As the effects of surgery diminish and patients begin to manage their weight independently, their psychological wellbeing and functioning may evolve in ways that differ from the trajectories presented in this study. The present findings could be a useful source for hypothesis generation regarding the longitudinal associations and pathways between psychological characteristics and weight outcomes. Finally, future studies could investigate whether psychological support or intervention, for example weight management training incorporating elements of self-compassion, offered pre- or postoperatively can help change the course of recovery trajectory.

## Conclusion

This prospective study utilized LCGA to investigate longitudinal changes in weight, HR-QoL, and depression over a 24-month period following BMS. Three distinct trajectories emerged for BMI and HR-QoL, characterized by initial improvement and subsequent continued improvement, stabilization, or deterioration. A noteworthy finding was the identification of depressive symptom patterns, with the majority of patients showing low and improving or consistently low levels, while approximately 20% experienced a deterioration in depression. Notably, considerable heterogeneity in post-bariatric surgery outcomes was observed. Importantly, patients with the most favourable weight loss trajectory did not necessarily exhibit psychologically favourable outcomes, and preoperative characteristics did not reliably identify post-surgical BMI trajectories. This underscores the complexity of predicting surgical success based on preoperative psychological status. Clinicians should incorporate monitoring both wellbeing and depression long term after BMS for effective patient care. Problems could arise after the surgery, thus individualized care could benefit patients. The study findings emphasize the significance of incorporating psychological health as an essential component of clinical outcomes.

Two important take-home messages emerged from this investigation: (1) predicting post-surgical BMI trajectories from preoperative psychological characteristics is challenging if not impossible due to the large variation in patient profiles, and (2) while an average weight loss patient seems to be doing well in terms of their psychological health, patients following the more ‘successful’ BMI trajectory may be more likely to struggle with their psychological health. These findings encourage clinicians to be mindful of patients following the two most divergent BMI trajectories.

## Data Availability

Deidentified study data will be shared upon reasonable request by contacting the corresponding author. The full study protocol is published and available to the public (van Olden et al., 2021).

## Declarations

### Clinical trial registration

BARIA- study: Netherlands Trial Register: NL5837 (NTR5992). https://onderzoekmetmensen.nl/en/trial/22589. Date: 13-07-2016. Diabar- study: ClinicalTrials.gov identifier (NCT number): NCT03330756. Date of registration: 2017–10-30.
